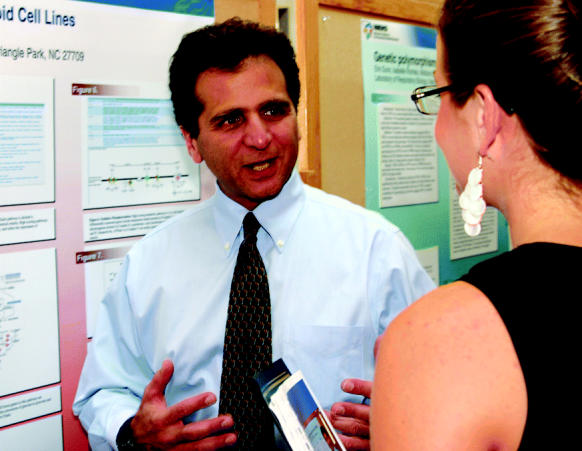# Training the Next Generation

**DOI:** 10.1289/ehp.113-a578

**Published:** 2005-09

**Authors:** David A. Schwartz

**Affiliations:** Director, NIEHS and NTP, E-mail: schwartzd@niehs.nih.gov

Many factors go into achieving the highest-quality research: a clear vision, sufficient funding, and supportive facilities, to name just a few. But without question, the most critical factor is the human one. All great research starts with a creative idea, which comes from an individual or a group of people working together to solve a problem. The mission of the NIEHS is to understand how the environment influences the development and progression of human disease, and so it is imperative that we bring the best minds to bear on these problems. Recruitment, retention, and career development are challenges for every aspect of biomedical research, including environmental health sciences. Thus, as we reconceptualize the opportunities and challenges in our field, we at the NIEHS will critically evaluate our approach to research training and career development. To produce the best science, we must nurture the best scientists.

Training and career development in environmental health sciences is clearly one of our most profound responsibilities. However, this is not a simple process. Although research training and career development are the most obvious in the early stages of mentored research, this process should be viewed as a continuum from initial recruitment and engagement in biomedical research through the initial and subsequent discoveries that lead to an established and productive career in environmental health sciences. Our institute needs to establish a clear path to support the development of young scientists throughout their progression from student to senior investigator.

We at the NIEHS will critically evaluate our approach to research training and career development. To produce the best science, we must nurture the best scientists.

The first step in this progression is engagement in scientific research. Students at the high school and college levels must be introduced to the field of environmental health sciences, shown its relevance and potential for understanding human disease and solving the real-world problems that affect all of us, and provided both the opportunity and the encouragement to enter into the sometimes daunting realm of scientific research. To accomplish this, we will use and expand the tools we have—such as summer internship programs, undergraduate work–study programs, partnerships with professional and educational societies, and outreach through publications—to create an open door to welcome the best and brightest young minds to our field.

And we must realize that engagement in biomedical research is just the first step. In order to retain the best scientists in our field, we must create an environment in which young scientists can become ever more excited by their work, where they feel supported both intellectually and personally, and where they have the flexibility to move seamlessly within and outside of our intramural and extramural scientific communities.

Given the breadth of environmental health sciences, interdisciplinary research is an absolute necessity, especially in our training programs. This range of diversity creates an enormous opportunity to bring together teams of scientists with different backgrounds, skills, and ideas to more effectively tackle today’s critical problems in environmental health. An interdisciplinary approach will undoubtedly lead to more profound achievements in biomedical research, which will then translate into more substantial advances in human health. Our young scientists need to be trained in interdisciplinary research so that they can more effectively work with other scientists to ask and answer the toughest questions in environmental health sciences.

In addition, we must work with budding scientists at critical junctures in their careers, such as between college and graduate school or between mentored and independent research, to facilitate these transitions so that the movement into or retention in environmental health sciences becomes a natural progression that is supported by the NIEHS. This could be accomplished by a combination of mentoring as well as established and evolving extramural programs including career transition awards, loan repayment programs, and awards for new investigators to support not just research costs but also the startup costs for new laboratories.

However, career development is fundamentally dependent on mentorship. When I think back to my own career development, I am astonished by the profound impact of my outstanding mentors. For instance, throughout the past 15 years, Gary Hunninghake, professor of medicine at The University of Iowa and one of my many mentors, has continually prodded and guided me to the next level of achievement through independent research support, scientific program development, and encouragement to become the director of the NIEHS. Gary and other great mentors instill in their trainees a passion for their work that embraces creativity, individuality, dedication, fearlessness, and personal balance. The NIEHS needs to improve it’s ability to develop and support such mentors.

And while investing in the training of researchers at our own institute, we should renew and expand our efforts to become a central location for the continued education and training of scientists from around the world. NIEHS scientific director Lutz Birnbaumer recently suggested that we seek to establish the NIEHS as a “campus of learning,” somewhat in the model of Cold Spring Harbor or the Jackson Laboratory, whereby we would offer continuing education and training in environmental health sciences through programs such as summer fellowships, short courses, and longer sabbatical opportunities. Such programs would be taught by a combination of NIEHS and outside scientists who would bring a wide variety of skills, ideas, perspectives, and expertise.

One definition of “institute” is a place for instruction. The process of strategic planning under way at the NIEHS will aid us in focusing on how we can achieve our future research and training goals as we strive to fulfill the promise of our name and become a true institute for scientific learning and discovery.

## Figures and Tables

**Figure:**